# Cyanidin-3-*O*-Glucoside Regulates the M1/M2 Polarization of Microglia via PPARγ and Aβ42 Phagocytosis Through TREM2 in an Alzheimer’s Disease Model

**DOI:** 10.1007/s12035-022-02873-9

**Published:** 2022-06-07

**Authors:** Sanjay, Jae-Ho Shin, Miey Park, Hae-Jeung Lee

**Affiliations:** 1 Department of Food Science and Biotechnology, College of BioNano Technology, Gachon University, Seongnam-si 13120, Gyeonggi-do, Korea; 2 Department of Biomedical Laboratory Science, Eulji University, Gyeonggi-do 461-713, Seongnam-si, Republic of Korea; 3 Department of Food and Nutrition, College of BioNano Technology, Gachon University, Seongnam-si 13120, Gyeonggi-do, Korea; 4 Institute for Aging and Clinical Nutrition Research, Gachon University, Seongnam-si 13120, Gyeonggi-do, Korea

**Keywords:** Alzheimer’s disease, Neuroinflammation, Anthocyanin, Cyanidin-3-*O*-glucoside, Anti-inflammation, M1/M2 shift

## Abstract

Microglial polarization plays an essential role in the progression and regression of neurodegenerative disorders. Cyanidin-3-*O*-glucoside (C3G), a dietary anthocyanin found in many fruits and vegetables, has been reported as an antioxidant, anti-inflammatory, and antitumor agent. However, there have been no reports on whether C3G can regulate the M1/M2 shift in an Alzheimer’s disease model. We attempted to investigate the effects of C3G on M1/M2 polarization and the mechanism to regulate anti-inflammation and phagocytosis, both in vitro and in vivo. HMC3 cells were treated with β-amyloid (Aβ42) in the presence or absence of 50 μM C3G for different time intervals, and APPswe/PS1ΔE9 mice were orally administered 30 mg/kg/day of C3G for 38 weeks. The in vitro data revealed that C3G could shift the M1 phenotype of microglia to M2 by reducing the expression of M1-specific markers (CD86 and CD80), inflammatory cytokines (IL-Iβ, IL-6, TNF-α), reactive oxygen species, and enhancing the expression of M2-specific markers (CD206 and CD163). The APPswe/PS1ΔE9 mice results were consistent with the in vitro data, indicating a significant reduction in inflammatory cytokines and higher expression of M2-specific markers such as CD206 and Arg1 in C3G-treated Alzheimer’s disease model mice. Additionally, C3G was found to upregulate PPARγ expression levels both in vitro and in vivo, whereas a PPARγ antagonist (GW9662) was found to block C3G-mediated effects in vitro. In this study, we confirmed that C3G could regulate microglial polarization by activating PPARγ and eliminating accumulated β-amyloid by enhancing Aβ42 phagocytosis through the upregulation of TREM2.

## Introduction

Alzheimer’s disease (AD) is a progressive neurodegenerative brain disorder that affects memory and cognitive functions and challenges an individual’s ability to think, communicate clearly, and carry out day-to-day tasks. It accounts for two-thirds of all dementia cases, with 50–70% of people with dementia afflicted with AD. AD is characterized by the presence of β-amyloid (Aβ) plaques and neurofibrillary tangles (NFTs) within the brain. Aβ plaques develop as a consequence of the faulty cleavage of amyloid precursor protein (APP), whereas NFTs are produced via the hyper-phosphorylation and disorganization of tau proteins [1].

Recent studies have identified neuroinflammation as the primary cause of AD progression [2]. Microglia, the brain macrophages, play an essential role in countering the extracellular accumulation of Aβ plaques by dividing them into two distinct phenotypes. The M1 phenotype contributes to neurotoxicity, oxidative stress, and neuronal and synaptic damage. In contrast, the M2 phenotype plays an essential role in regulating neuroinflammation by clearing cell debris and misfolded proteins and providing support for neuroregeneration. The M1 phenotype of microglia produces pro-inflammatory cytokines such as IL-1β, TNF-α, and IL-6 and expresses the M1-specific markers CD86 and CD80 [3–5]. However, cells with the M2 phenotype release anti-inflammatory cytokines such as IL-10 and IL-4 and the M2-specific anti-inflammatory markers CD206 and CD163 [6–8]. Additionally, reactive oxygen species (ROS) play a pivotal role in the pathogenesis of AD and are responsible for promoting a number of harmful effects, including damage to cellular DNA, lipids, and proteins [9–11].

Anthocyanins are a class of polyphenolic flavonoids found in the fruits, flowers, leaves, and seeds of a range of plants, including cherry, blueberry, blackcurrant, mulberry, black soybean, and honeyberry [12, 13]. These compounds have attracted considerable attention in recent years, owing to their potential therapeutic properties, with beneficial effects such as those associated with neuroinflammation, excitotoxicity, and protein homeostasis [14, 15]. Cyanidin-3-*O*-glucoside (C3G) is a flavonoid anthocyanin studied as an ant oxidative, anti-inflammatory, and antitumor agent [16–19]. It has also been found to have several beneficial effects in treating different diseases and disorders, such as non-alcoholic fatty acid liver disease, cardiovascular diseases, and neurological disorders [20–23].

Peroxisome-proliferator activated receptors (PPARs) are ligand-activated nuclear receptors that regulate different biological pathways, including those associated with cell proliferation, development, metabolism, reproduction, and inflammation. These receptors can be sub-divided into three isoforms, namely, PPAR-α, PPAR-γ, and PPAR-δ, which are distributed in different tissues, in which they perform various biological functions [24]. Although studies have reported that the beneficial effects of C3G are mediated via PPARs [25, 26], there have been no reports on the role of PPARγ in the C3G-mediated neuroprotective effects in Aβ42-treated human microglial HMC3 cells. Additionally, whereas it has been established that the impact of certain flavonoids on M2 polarization is mediated via the activation of PPARγ, no studies have been reported to date that has examined the effects of C3G in mediating microglial phenotypes via PPARγ.

The triggering receptor expressed on myeloid cells (TREM2) is a receptor found on the surface of microglial cells that plays an essential role in the phagocytosis of Aβ. The progression of AD has been reported to be associated with alterations in the expression of TREM2 and poor phagocytosis of Aβ by microglial cells [27–30]. Moreover, recent studies have revealed that TREM2 functions as an M2-specific microglia marker [31–33], and C3G has been reported to clear Aβ25-35 and inhibit Aβ40 fibrillogenesis in vivo [34]. To the best of our knowledge, however, no previous studies have examined the effects of C3G on TREM2 expression and Aβ42 phagocytosis in human microglial cells.

In this study, we investigated the effects of C3G in regulating the M1/M2 shift of microglia and Aβ42 phagocytosis in Aβ42-treated human microglial cells and sought to determine the role of PPARγ and TREM2 in the C3G-mediated effects.

## Material and Methods

### Materials

The HMC3 (ATCC® CRL-3304™) cell line used in this study was obtained from the American Type Culture Collection (ATCC, Manassas, USA). C3G (Cat. No. CFN99740) was purchased from ChemFaces (Wuhan, Hubei, China). Specific antibodies against CD86 (sc-19617), CD206 (sc-58986), CD163 (sc-20066), and TREM2 (sc-373828) were purchased from Santa Cruz Biotechnology (Dallas, TX, USA). Phycoerythrin (PE)-conjugated CD80 antibody (12–0809-42) was purchased from eBioscience (San Diego, CA, USA). TREM2 blocking antibody (MAB17291-100) was purchased from Bio-techne (Minneapolis, MN, USA). PE-conjugated secondary antibody (ab97024) and Aβ42 peptide (ab120301) were purchased from Abcam (Cambridge, UK). The PPARγ antagonist (GW9662; Cat. no M6191) was purchased from Sigma (St. Louis, MO, USA). Dulbecco’s modified Eagle’s medium (DMEM), trypsin, and fetal bovine serum (FBS), antibiotic–antimycotic solution, were supplied by Thermo Fisher (San Jose, CA, USA).

### Cell culture and Treatments

The HMC3 immortalized human microglial cell line was grown optimally in DMEM supplemented with 10% heat-inactivated FBS and 1% antibiotic–antimycotic solution and maintained in a 5% CO_2_ humidified incubator at 37 °C. Both the β-amyloid and C3G were prepared in DMSO, so DMSO was used as a control in our study.

### Cell Viability Assay

HMC3 cells were seeded in 96-well plates (1 × 10^4^ cells/well) and allowed to adhere and grow for 24 h. After stabilization for 24 h, the cells were exposed to different concentrations (0.1, 1, 2, and 4 μM) of Aβ42 for 24 h and co-treated with 1 μM of Aβ42 and C3G (25, 50, 100, 200 μM) for 24 h incubation under a 5% CO_2_ atmosphere. Cell viability was assessed using a Cell Counting Kit-8 assay (Dojindo Molecular Technologies, Rockville, MD, USA), as recommended by the manufacturer. The absorbance was measured at 450 nm using a microplate reader (BioTek Inc., Winooski, VT, USA).

### Annexin V-FITC Apoptosis Detection Assay

Annexin V-FITC apoptosis detection assay kit (APOAF-20TST) purchased from Sigma (St. Louis, MO, USA) allows flow cytometry–based determination of apoptotic cells. The assay involves two significant components: annexin V conjugated with fluorescein isothiocyanate (FITC) to label phosphatidylserine sites on the membrane surface and propidium iodide (PI) to label the cellular DNA in necrotic cells where the cell membrane has been totally compromised. The combination of annexin V and PI allows the differentiation of viable cells (annexin V negative, PI negative), early apoptotic (annexin V positive, PI negative), and necrotic cells (annexin V positive, PI positive). In brief, HMC3 cells were treated with 1 μM of Aβ42 and co-treated with 1 μM of Aβ42 and 50 μM of C3G for 24 h and harvested. Cells were washed with DPBS, and each group, including the control, was divided into triplicates. For each sample, cells were suspended into 500 µL of 1X binding buffer containing 5 µL of annexin V-FITC and 5 µL of PI in the dark for 10 min at room temperature. Flow cytometry analysis was performed to detect apoptotic and viable cells using an FC500 MLP cytometer (Beckman Coulter Inc., Fullerton, CA, USA).

### RNA Preparation and Real-Time PCR

Having treated the HMC3 cells with the 1 μM of Aβ42 for different time intervals (1, 3, 6, and 12 h), and co-treated with 1 μM of Aβ42 and 50 μM of C3G for 3 h, total RNA was extracted using an RNA extraction kit (iNtRON Biotechnology, Gyeonggi-do, Korea) according to the manufacturer’s instructions. In total, 50 ng of RNA was reverse transcribed to complementary DNA using a PCR (TaKaRa Bio, Kusatsu, Shiga, Japan). RT-PCR was performed with TB Green (TaKaRa Bio, Kusatsu, Shiga, Japan) using an ABI QuantStudio3 PCR system (Applied Biosystems, Foster City, CA). All reactions were performed in triplicate. PCR amplification of target genes in human microglial cells and mouse cortex tissue was performed using specific primer sets, the sequences of which are listed in Table 1. Gene expression was normalized using human or mouse β-actin genes as endogenous controls. Cells were plated in 96-well plates (1 × 10^4^ cells/well) and allowed to adhere and grow for 24 h. After stabilization for 24 h, the cells were exposed to different concentrations of the desired compounds and incubated for different time intervals under a 5% CO_2_ atmosphere. Cell viability was assessed using a Cell Counting Kit-8 assay, with absorbance being measured at 450 nm using a microplate reader.

**Table 1:** The sequences of primers used to amplify target human and mouse genes

Species	Target gene	Forward primer	Reverse primer	Reference
**Human**	**TNF-α**	TGAGCACTGAAAGCATGATCC	GGAGAAGAGGCTGAGGAACA	[35]
	**IL-6**	GACCCAACCACAAATGCCAG	GAGTTGTCATGTCCTGCAGC	[36]
	**IL-1β**	GGGATAACGAGGCTTATGTGC	AGGTGGAGAGCTTTCAGTTCA	[37]
	**β-actin**	CTCTTCCAGCCTTCCTTCCT	AGCACTGTGTTGGCGTACAG	[38]
**Mouse**	**Arg-1**	CTTGCGAGACGTAGACCCTG	TCCATCACCTTGCCAATCCC	[39]
	**CD206**	TCAGCTATTGGACGCGAGGCA	TCCGGGTTGCAAGTTGCCGT	[40]
	**CD86**	ACGATGGACCCCAGATGCACCA	GCGTCTCCACGGAAACAGCA	[31]
	**PPARγ**	TTTTCAAGGGTGCCAGTTTC	AATCCTTGGCCCTCTGAGAT	[41]
	**TREM2**	TGGGACCTCTCCACCAGTT	GTGGTGTTGAGGGCTTGG	[42]
	**TNF-α**	ACCGCAACAACGCCATCTAT	GTATCAGTGGGGGTCAGCAG	[43]
	**IL-6**	AGACAAAGCCAGAGTCCTTCA	GGTCCTTAGCCACTCCTTCTG	[44]
	**IL-1β**	CACAGCAGCACATCAACAAG	GTGCTCATGTCCTCATCCTG	[44]
	**β-actin**	CTGTCCCTGTATGCCTCTG	ATGTCACGCACGATTTCC	[38]

*TNF-α* tumor necrosis factor-alpha, *IL-6* interleukin 6, *IL-1β* interleukin 1β, *Arg-1* arginase 1, *CD206* cluster of differentiation 206, *CD86* cluster of differentiation 86, *PPARγ* peroxisome proliferator-activated receptor γ, *TREM2* triggering receptors expressed on myeloid cells

### Flow Cytometry

HMC3 cells were treated with 1 μM of Aβ42, and co-treated with 1 μM of Aβ42 and 50 μM of C3G for 24 h. Cell surface markers were stained with primary anti-mouse CD86 MAb, anti-mouse CD206 MAb, anti-mouse CD163 MAb, and anti-mouse TREM2 MAb for 30 min at 4 °C. After washing twice with phosphate-buffered saline (PBS), cells were treated with secondary PE-conjugated goat anti-mouse IgG H&L antibody for 30 min at 4 °C and washed twice with PBS. For observing cell surface expression of CD80, PE-conjugated CD80 antibody was used for staining at 4 °C for 30 min. Following further washes, the cells were resuspended in 0.4 mL of PBS and analyzed to express cell surface proteins using a Cytomics FC500 MLP cytometer (Beckman Coulter Inc., Fullerton, CA, USA).

For observing Aβ42-phagocytosis through flow cytometry, cells were treated with 1 μM of Aβ42, and co-treated with 1 μM of Aβ42 and 50 μM of C3G for 24 h. Cells were harvested and suspended in 4% paraformaldehyde for 10 min, washed and treated with 0.1% Triton-X for 10 min followed by treatment with anti-β-amyloid, 1–42 antibody for 30 min at 4 °C. Intracellular uptake of Aβ42 was observed by treating the cells with PE-conjugated goat anti-mouse IgG H&L antibody for 30 min and analyzed using cytometer.

### 2′,7′–Dichlorofluorescein Diacetate Assay

Dichloro-dihydro-fluorescein diacetate (DCFH-DA) is a membrane-permeable compound that can be enzymatically converted to the highly fluorescent compound 2′,7′-dichlorofluorescein (DCF) in the presence of ROS. HMC3 cells were grown in DMEM supplemented with 10% FBS and 1% penicillin/streptomycin solution at 37 °C in a 5% CO_2_ atmosphere, with the medium being changed every other day. Approximately 5 × 10^4^ cells were seeded in black 96-well plates. After incubating for 24 h in a cell incubator, the cell medium was removed, followed by the treatment of 1 μM of Aβ42, and co-treatment of 1 μM of Aβ42 and 50 μM of C3G for 24 h. The medium was washed out, and the cells were treated with 2′,7′-DCFH-DA for 30 min.

Fluorescence was monitored on a microplate reader with excitation and emission wavelengths of 488 nm and 525 nm, respectively, using a microplate reader and also examined under a fluorescence microscope. The results are expressed as a percentage relative to that of DFC fluorescence in the control cells.

### Immunofluorescence

HMC3 cells were treated with the 1 μM of Aβ42, and co-treated with 1 μM of Aβ42 and 50 μM of C3G for 24 h and washed with PBS, after which they were treated with 4% paraformaldehyde for 10 min and washed three times with PBS. After that, the cells were permeabilized with 0.1% Triton X-100 for 10 min and washed three times with PBS. After permeabilization, the cells were blocked with 5% bovine serum albumin in PBST (PBS + 0.1% Tween-20). The cells were then stained with the primary anti-β-amyloid, 1–42 antibody by incubating overnight. Having been washed three times, the cells were incubated with fluorescein isothiocyanate–conjugated secondary antibodies for 30 min and washed. The cell nuclei were stained with 1 mg/mL 4′,6-diamidino-2-phenylindole for 5 min, washed three times with PBS, and analyzed using a fluorescence microscope (Nikon, Tokyo, Japan).

### Animal Diet and Tissue Sample Preparation

Nine-month-old female AD mice (B6C3-Tg (APPswe/PS1ΔE9 85Dbo/Mmjax)) were purchased from the Jackson Laboratory, and age-matched 9-month-old non-transgenic mice (C57BL/6 J Jms) were obtained from SLC (Hamamatsu-shi, Shizuoka, Japan). The AD and non-transgenic mice were acclimatized and fed normal food pellets.

After acclimating for 2 weeks, the mice were randomized into the following three groups: non-transgenic mice (NC), AD mice treated with PBS (APPswe), and AD mice orally administered 30 mg/kg/day C3G (APPswe_C3G). The mice had ad libitum access to food and water for 38 weeks. Food intake was monitored twice per week, and body weight was measured once a week. All animal experiments were conducted in accordance with the Ministry of Food and Drug Safety (MFDS) Guidelines for Care and Use of Laboratory Animals and were approved by Eulji University (approval no. EUIACUC 20–13). All efforts were made to minimize the number of animals used and their suffering. At the end of the experimental period, the mice were fasted overnight and anesthetized with CO_2_. Blood was collected by cardiac puncture, and the brains were removed. The cortex was dissected from the right hemisphere, and total RNA was isolated using an RNA extraction kit (iNtRON Biotechnology, Gyeonggi-do, Korea).

### Statistical Analysis

The experiments were conducted at least three times, and all data are shown as mean ± standard deviation (SD). Significant differences among groups were analyzed by GraphPad Prism 5.03 (GraphPad Sofware, San Diego, CA, USA). One-way ANOVA and Tukey’s post hoc tests were used to analyze the results. Differences were considered statistically significant at *P* < 0.05.

## Results

**Fig. 1:**
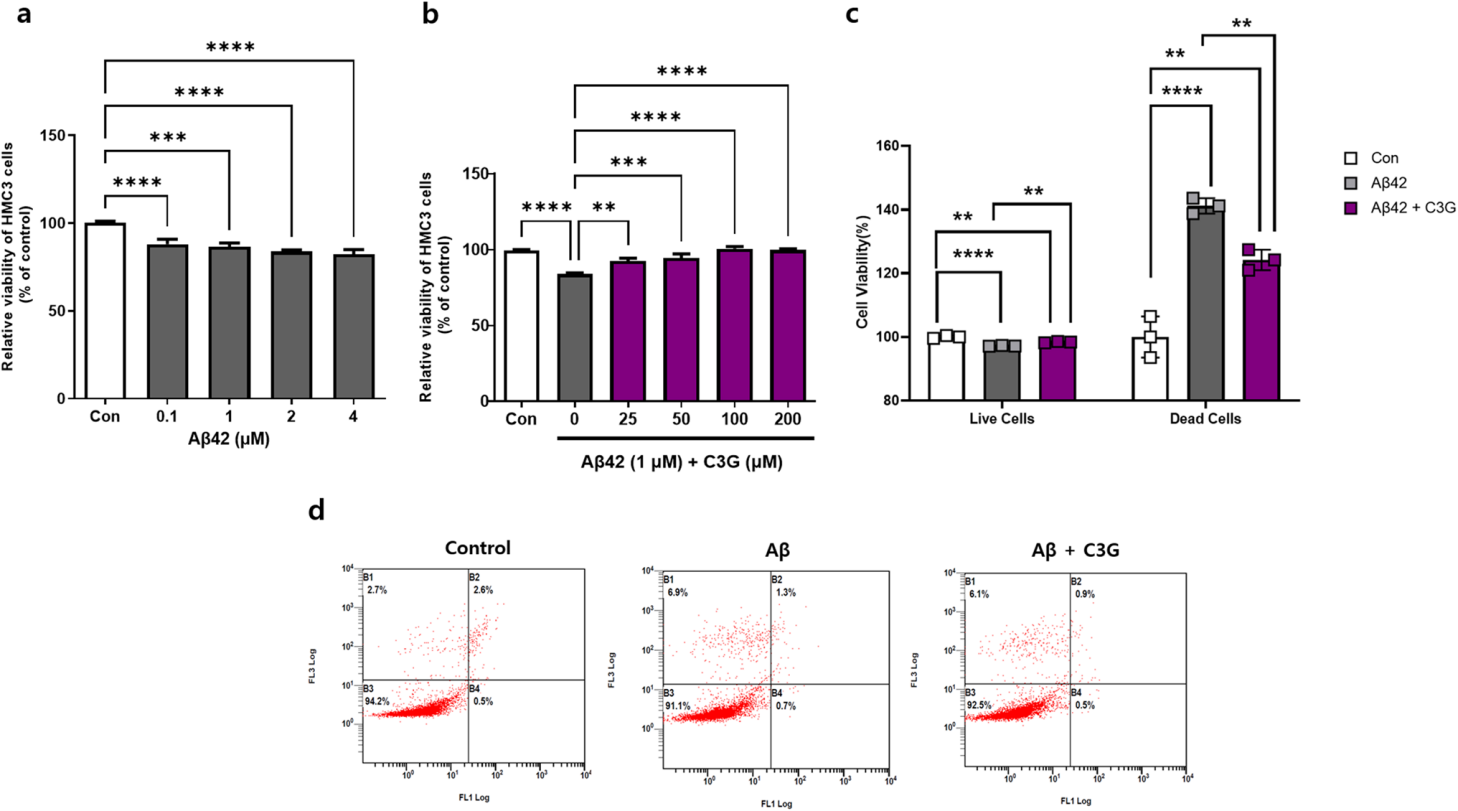
Cyanidin-3-O-glucoside (C3G) protects HMC3 cells from β-amyloid (Aβ42)-induced cytotoxicity. **a** Human microglial HMC3 cells were treated with different concentrations (0.1, 1, 2, and 4 μM) of Aβ42 for 24 h. **b** HMC3 cells were co-treated with 1 μM Aβ42 and different concentrations (25, 50, 100, and 200 μM) of C3G, and cell viability was observed using CCK assay. **c**–**d** HMC3 cells were treated with 1 μM Aβ42 and co-treated with 1 μM Aβ42 and 50 μM C3G and analyzed for apoptotic cells through annexin-V apoptosis assay kit. **p < 0.01, ***p < 0.001, and **** *p* < 0.0001

**Fig. 2:**
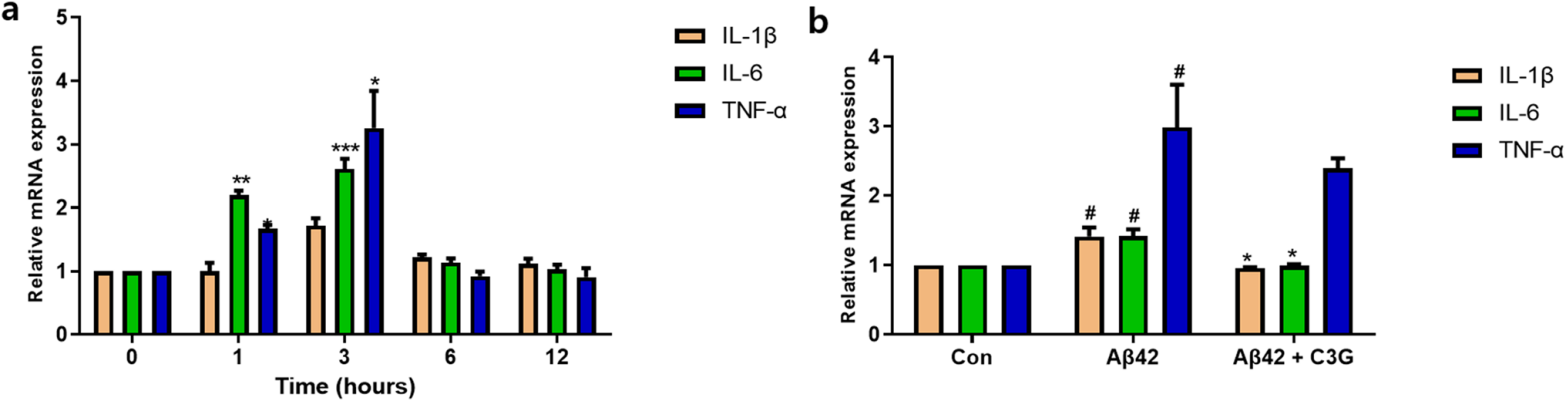
C3G downregulates the expression of Aβ42-induced pro-inflammatory cytokines in HMC3 cells. **a** HMC3 cells were treated with 1 μM Aβ42 for different time intervals (0, 1, 3, 6, and 12 h), and the mRNA expression of pro-inflammatory cytokines (IL-1β, TNF-α, and IL-6) was analyzed by RT-PCR. **b** Having established that maximal expression of the cytokines was obtained with a 3-h incubation, we examined the effects of 50 μM C3G in a co-treatment with 1 μM Aβ42 for 3 h on cytokine mRNA expression levels by RT-PCR. **a** *p < 0.05, **p < 0.01, and ***p < 0.001 with 0 group. **b** # *p* < 0.05 with Con; * *p* < 0.05 and ** *p* < 0.01 with Aβ42

To assess the effects of Aβ42 on HMC3 cells, the cells were treated with different concentrations (0.1–4 μM) of Aβ42 for 24 h. Aβ42 was accordingly found to induce cytotoxicity in HMC3 cells in a concentration-dependent manner, with a 1-μM concentration promoting a 20% reduction in cell viability after 24 h. We thus used Aβ42 at this concentration in further experiments (Fig. 1a). Having established a suitable cytotoxic concentration of Aβ42, HMC3 cells were treated with 1 μM of Aβ42 in the presence of different concentrations (25–200 μM) of C3G for 24 h to determine the effects of C3G on Aβ42-induced cytotoxicity. In line with expectations, we found that C3G protected the cells from Aβ42-induced cytotoxicity in a concentration-dependent manner, with the identified optimum concentration of 50 μM being used in further experiments (Fig. 1b). We repeated a similar experiment using the Annexin-V apoptosis assay kit to confirm our results. After giving the desired treatments, the percentage of viable and apoptotic cells was observed. It was found that the number of viable cells was decreased, and dead cells were increased significantly in the Aβ42-treated group compared to the control. However, in the co-treatment group of Aβ42 and C3G, cells retained their viability, and the number of dead cells also decreased significantly (Fig. 1c–d). Having assessed the cytoprotective effects of C3G, we went on to examine the anti-inflammatory effects of C3G. HMC3 cells were initially treated with 1 μM Aβ42 for different time intervals (0, 1, 3, 6, and 12 h) to determine the length of Aβ42 incubation necessary to induce optimal mRNA expression of common pro-inflammatory cytokines (IL-1β, TNF-α, and IL-6) for induction of an Alzheimer’s-like pathology. We accordingly found that incubation with 1 μM of Aβ42 for 3 h promoted a significant increase in the expression of pro-inflammatory cytokine mRNAs, with IL-1β, TNF-α, and IL-6 showing 2.45-, 2.6-, and 2.45-fold increases, respectively, compared with the control (Fig. 2a).

After determining the optimal incubation time, we repeated the experiment in the presence or absence of 50 μM C3G. Compared with the Aβ42-treated group, the levels of IL-1β and IL-6 mRNA were found to show a significant return to normal levels in the group receiving the combined Aβ42 and C3G treatment. In contrast, those of TNF-α showed a slight non-significant reduction (Fig. 2b). These findings accordingly indicated the potential of C3G to block the production of pro-inflammatory cytokines.

Having determined the effects of C3G in modulating Aβ42-induced pro-inflammatory cytokine production, we examined the effects of C3G for shifting the M1 phenotype of microglia to an M2 phenotype. To this end, HMC3 cells were treated with 1 μM Aβ42 alone or in combination with 50 μM C3G for 24 h. The effects were assessed by determining cell surface protein expression levels of markers specific for M1 (CD86 and CD80) and M2 (CD206 and CD163) using flow cytometry.

**Fig. 3:**
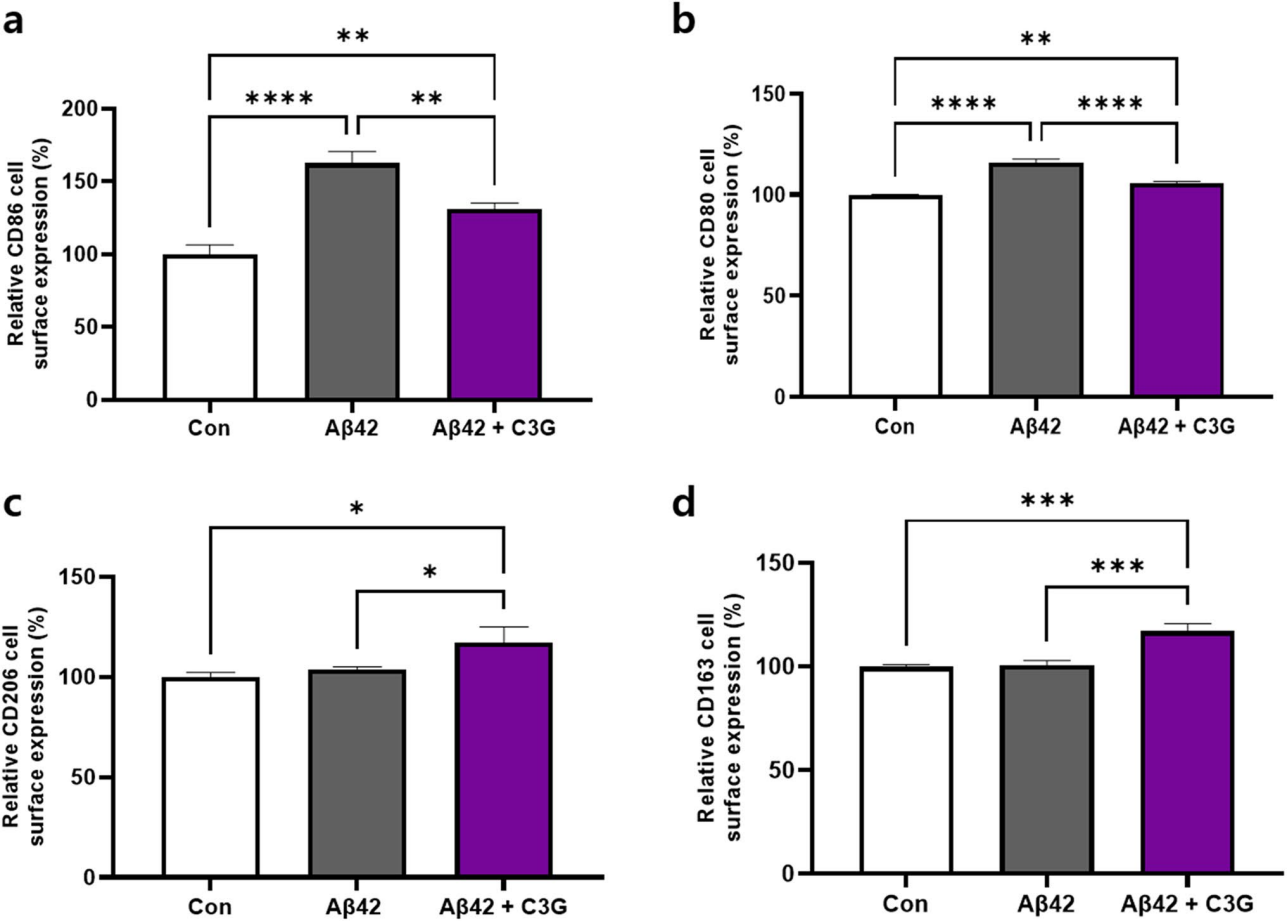
C3G regulates microglial polarization in Aβ42-induced HMC3 cells. Cells were treated with 1 μM Aβ42 and co-treated with 1 μM Aβ42 and 50 μM C3G for 24 h. **a**–**b** Relative cell surface protein expression of the M1-specific markers CD86 and CD80 and **c**–**d** M2-specific markers CD206 and CD163 was determined using flow cytometry. *p < 0.05, **p < 0.01, ***p < 0.001, and ****p < 0.0001

We found that the cell surface expression of CD86 and CD80 proteins in the Aβ42-treated group was significantly upregulated, by up to 62% and 15%, respectively, compared with the control, but were downregulated in the Aβ42 and C3G co-treated group. In contrast, cell surface expression of the M2-specific markers CD206 and CD163 was virtually unaffected in the Aβ42-treated group. In contrast, significantly upregulated expression of up to 17% and 17%, respectively, was detected in the Aβ42 and C3G co-treated groups (Fig. 3a–d). These results thus indicate the potential of C3G to shift the M1 phenotype of microglia to an M2 phenotype.

PPARγ has previously been found to mediate a number of the beneficial effects of flavonoids and has also been reported involved in macrophage polarization. We thus proceeded to examine the effects of C3G on the protein expression of PPARγ in β-amyloid-induced HMC3 cells and determine the role of PPARγ in shifting the phenotype of microglia.

**Fig. 4:**
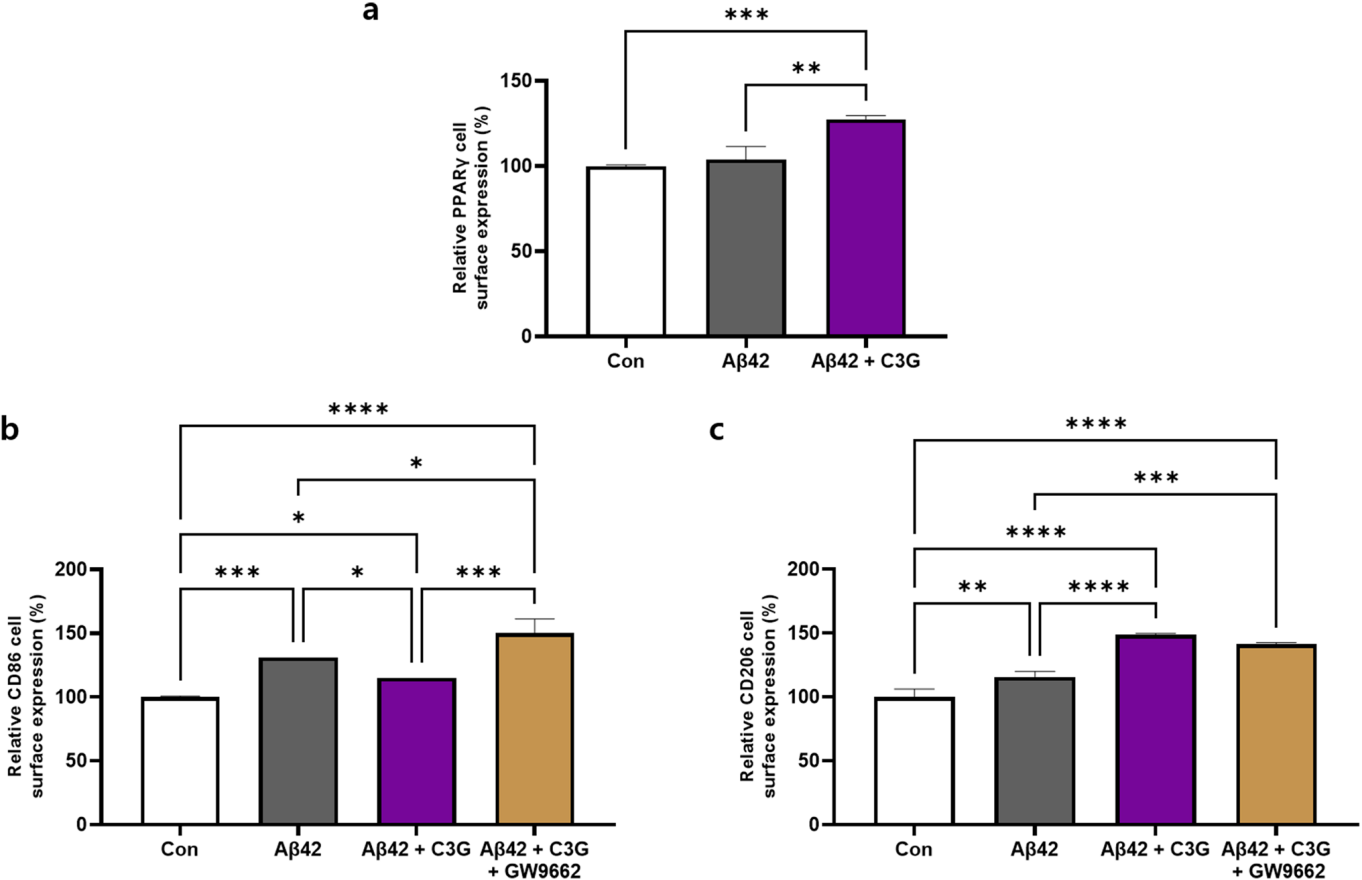
C3G regulates microglial M1/M2 polarization via PPARγ. **a** HMC3 cells were treated with 1 μM Aβ42 or co-treated with 1 μM Aβ42 and 50 μM C3G. The cell surface protein expression of PPARγ was observed using flow cytometry. **b**–**c** HMC3 cells were pretreated with 10 μM GW9662 for 30 min, followed by co-treatment with 1 μM Aβ42 and 50 μM C3G for 24 h. Expression of the M1-specific marker CD86 and M2-specific marker CD206 was determined using flow cytometry. *p < 0.05, **p < 0.01, ***p < 0.001, and ****p < 0.0001

HMC3 cells were initially treated with 1 μM Aβ42 for 24 h in the presence or absence of 50 μM C3G, and cell surface expression of the PPARγ protein was determined using flow cytometry. We observed that Aβ42 had almost no effect on the expression of PPARγ, whereas a significantly upregulated expression (by 23%) was detected in cells co-treated with Aβ42 and C3G, thereby indicating the role of PPARγ in mediating the anti-inflammatory effects of C3G (Fig. 4a).

These experiments were subsequently repeated in the presence of a PPARγ antagonist (GW9662). HMC3 cells were initially pretreated with 10 μM GW9662 for 30 min, followed by post-treatment with Aβ42 and C3G for 24 h. We monitored the protein expression of the classically activated microglia-specific markers CD86 and CD206. We accordingly observed that the C3G-mediated downregulation of CD86 was significantly blocked in the presence of the PPARγ antagonist. Moreover, we detected the upregulated expression of this protein by up to 26%, 56%, and 40% compared with that in cells receiving the Aβ42, control, and Aβ42/C3G treatments, respectively (Fig. 4b).

Contrastingly, the expression of CD206 was found to be either virtually unaffected or non-significantly downregulated (Fig. 4c). Therefore, these results indicate that PPARγ was involved in mediating the display of M1 specific marker CD86 although not M2 marker CD206.

**Fig. 5:**
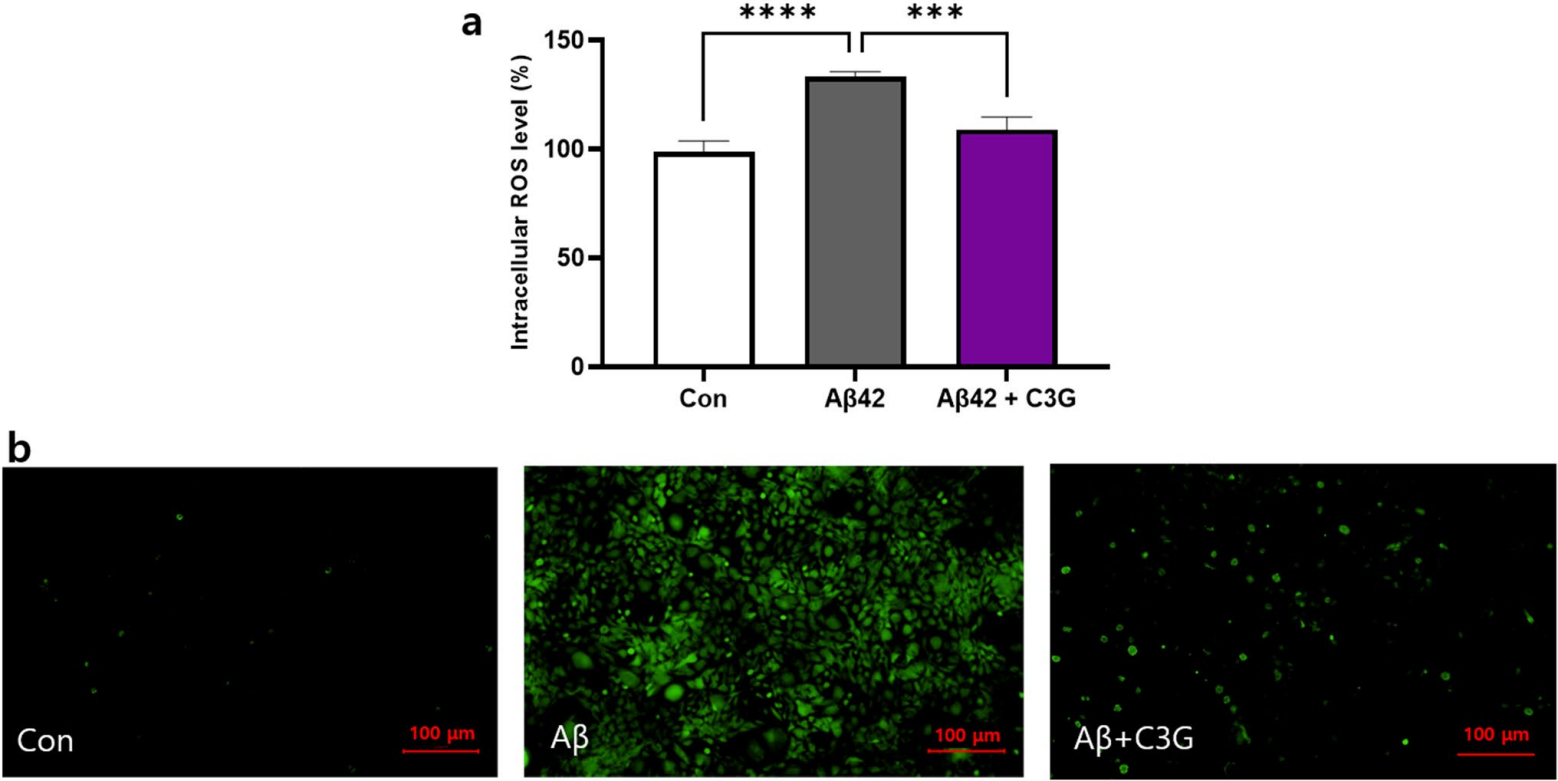
C3G attenuates Aβ42-induced reactive oxygen species (ROS) production. HMC3 cells were treated with 1 μM Aβ42 and co-treated with 1 μM Aβ42 and 50 μM C3G for 24 h. Intracellular ROS levels in Aβ42-induced HMC3 cells were determined using DCFDA assay through **a** microplate reader and **b** fluorescence microscopy. **p < 0.01 and ***p < 0.001. Scar bar is 100 μm

And to determine the anti-oxidative effects of C3G, HMC3 cells were treated with 1 μM Aβ42 in the presence or absence of 50 μM C3G for 24 h. ROS levels were determined using a DCFDA assay with microplate reader/fluorescence microscopy detection. We found that in cells treated with Aβ42 alone, there was a significant increase in ROS levels of up to 23% compared with that in the control, whereas ROS levels in cells receiving the combined Aβ42 and C3G treatment were observed to be decreased significantly (Fig. 5a). Similar results were obtained based on fluorescence microscopy observation, as indicated by the higher intensity of fluorescence signals detected in the Aβ42-treated cells compared with the control and Aβ42 and C3G co-treated cells (Fig. 5b).

**Fig. 6:**
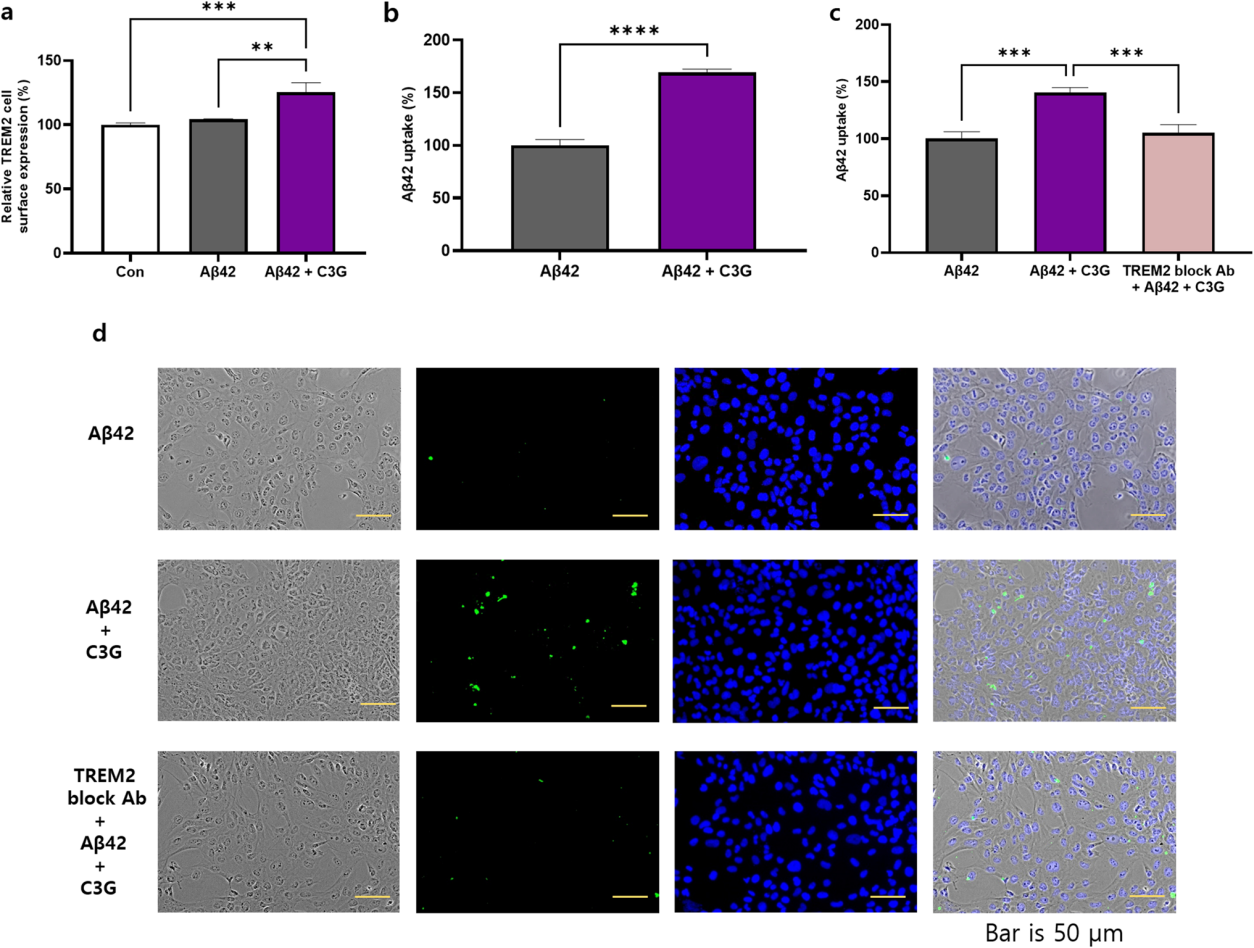
C3G mediates Aβ42-phagocytosis in Aβ42-treated HMC3 cells via triggering receptor expressed on myeloid cells (TREM2). HMC3 cells were treated with 1 μM Aβ42 and co-treated with 1 μM Aβ42 and 50 μM C3G for 24 h. **a** Cell surface protein expression of TREM2 was determined using flow cytometry. **b** Intercellular uptake of Aβ42 was determined using flow cytometry. **c**–**d** HMC3 cells were pretreated with 1 μM TREM2-blocking antibody for 1 h, followed by co-treatment with 1 μM Aβ42 and 50 μM C3G for 24 h. Intercellular uptake of Aβ42 was determine using flow cytometry and fluorescence microscopy (**d**). **p < 0.01, ***p < 0.001, and ****p < 0.0001. Scar bar is 50 μm

TREM2 is considered a common phagocytic receptor, and the findings of numerous studies have revealed altered patterns of TREM2 expression in AD pathology. In the present study, HMC3 cells were treated with Aβ42 (1 μM) for 24 h in the presence or absence of 50 μM C3G, and we subsequently examined the levels of cell surface TREM2 protein expression using flow cytometry. Cells co-treated with Aβ42 and C3G were found to be characterized by a significantly upregulated expression of TREM2 (up to 25%). In contrast, expression was unaffected in cells treated with Aβ42 alone (Fig. 6a).

To confirm our findings, we assessed whether C3G had any effect on the uptake of Aβ42 by HMC3 cells. HMC3 cells were initially treated with Aβ42 (1 μM) for 24 h in the presence or absence of 50 μM C3G, after which the cells were assessed by flow cytometry for intracellular Aβ42-uptake using an anti-Aβ42-antibody. We found that compared with the cells receiving the Aβ42 treatment, the presence of C3G enhanced the phagocytosis of Aβ42 by up to 69% (Fig. 6b).

Furthermore, to determine whether Aβ42 phagocytosis is mediated via TREM2, we used a TREM2 blocking antibody. Cells were initially treated with the TREM2 blocking antibody (1 µg/mL) for 1 h, followed by co-treatment with 1 μM Aβ42 and 50 μM C3G for 24 h. After that, intracellular Aβ42 uptake was examined based on flow cytometry analysis, which revealed that in cells treated with the TREM2 blocking antibody, the intracellular uptake of Aβ42 was significantly reduced compared with that observed in cells co-treated with C3G and Aβ42 and comparable to that observed in cells treated with Aβ42 alone. Similar observations were made using fluorescence microscopy. In cells co-treated with Aβ42 and C3G, the signal’s intensity was higher than that in cells treated with Aβ42 alone and TREM2 blocking antibody (Fig. 6c–d). These findings thus tend to indicate that TREM2 plays a role in the C3G-enhanced phagocytosis of Aβ42.

**Fig. 7:**
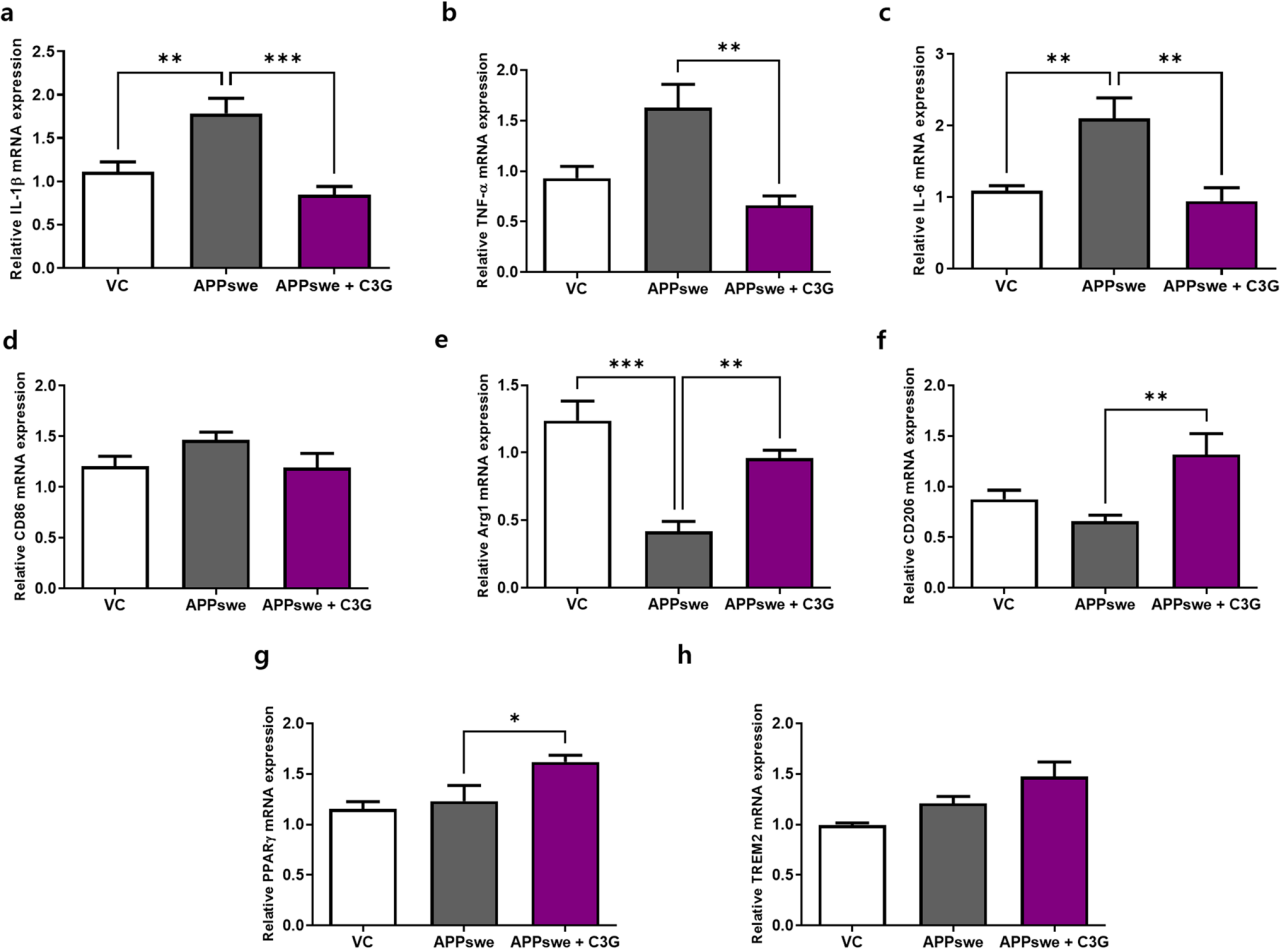
C3G attenuates neuroinflammation in the cortex of APPswe/PS1ΔE9 model mice. Mice were divided into three groups: non-transgenic mice (VC), AD mice administered PBS (APPswe), and AD mice administered 30 mg/kg/day C3G (APPswe + C3G). RNA was extracted from the cortex region of mouse brains and analyzed for mRNA expression of **a**–**c** inflammatory cytokines (IL-1β, TNF-α, and IL-6), **d** M1-specific marker (CD86), **e**–**f** M2-specific markers (Arg1 and CD206), and **g-h** PPARγ and TREM2 using RT-PCR. *p < 0.05, **p < 0.01, and ***p < 0.001

To confirm in vitro observation, we examined C3G-mediated anti-inflammatory effects in an AD (APPswe/PS1ΔE9) mouse model. The results revealed that C3G significantly suppressed IL-1β, TNF-α, and IL-6 gene expression in the cortex compared with that in APPswe mice, in which these cytokines showed 1.8, 1.5-, and 2-fold higher expression, respectively, compared with that in the vehicle control (Fig. 7a–c). Moreover, mRNA expression of the M1 macrophage marker CD86 decreased non-significantly, but M2 macrophage markers Arg-1 and CD206 were found to be significantly increased in the cortex of APPswe + C3G mice compared with that in APPswe mice (Fig. 7d–f). We also established that the mRNA expression of PPARγ, which was identified as a significant target in the regulation of inflammation in the cortex of APPswe + C3G mice, was significantly increased compared with that in VC and APPswe mice. Similarly, levels of TREM2 expression in the cortex of APPswe + C3G mice were also increased, although it was not significant (Fig. 7g–h).

## Discussion

AD is one of the significant health concerns of the present century and is recognized as the most common cause of dementia. It is an affliction from which an estimated 50 million people are believed to suffer worldwide [35]. It accounts for some two-thirds (50–70%) of all dementia cases, with those having pre-existing cardiovascular diseases, diabetes, or hypertension considered an exceptionally high risk of developing AD later in life. Although the exact cause of AD is yet to be conclusively identified, two factors that have been established to be closely linked with AD progression are the accumulation of Aβ plaques in the cerebral cortex and the neurofibrillary tangle formation of intra-nerve filamentous neurons associated with tau protein hyperphosphorylation [1]. These altered proteins and their excess accumulation within the brain alter normal brain cell functions. Along with neurons and astrocytes, brain-resident macrophages (microglia) are among the principal cells of the brain that play essential roles in dealing with extracellular stress [36]. Microglia are characterized by two distinct phenotypes; M1 type is the classically activated form associated with neurotoxicity, oxidative stress, synapses, and neuronal damage, and the other alternatively activated M2 or anti-inflammatory phenotypes play an important role in regulating inflammation, removing cell debris and misfolded proteins, and providing neurotrophic support for neuroregeneration [4, 37]. Neuroinflammation attributable to microglial cell activity has been reported as one of the main pathophysiological events involved in the progression of various neurodegenerative disorders, such as AD, Parkinson’s disease (PD), and multiple sclerosis [38]. In this regard, the findings of recent studies have provided evidence to indicate that microglial polarization (shifting the M1 phenotype to M2) is among the promising strategies for the treatment of a range of neurodegenerative disorders [39–44].

Dietary anthocyanins have attracted the attention of scientists on account of their established neuroprotective effects in the central nervous system [12]. These anthocyanins are absorbed as glycosides in rodents and humans, after which they cross the blood–brain barrier and localize within different regions of the brain, such as the cortex, cerebellum, hippocampus, and striatum [13]. C3G has been identified as the most abundant anthocyanin present in vegetables and fruits, and numerous studies have demonstrated the neuroprotective effects of C3G for AD, PD, and other neurodegenerative disorders in the recent years [25, 44–55]. However, there have been no studies that have examined C3G-mediated effects in Aβ-treated HMC3 cells. In the present study, we evaluated the impact of C3G in attenuating Aβ42-induced toxicity in HMC3 cells.

We initially assessed the concentration dependency of the effect of Aβ42 on HMC3 cells. Based on a 24-h incubation experiment, we found that Aβ42 can significantly induce cytotoxicity in HMC3 cells which was consistent with previous in vitro findings [25, 56]. We established that Aβ42 at a concentration of 1 µM would be ideal for performing further experiments. Moreover, we found that exposing cells to 1 µM Aβ42 in the presence of C3G had no appreciable effect on cell viability. The impact of C3G was found to be concentration-dependent, as has also been determined in previous studies [25, 52, 56]. Based on these observations, we established 50 µM to be a suitable working concentration for C3G, as this contributed to maintaining normal HMC3 viability in the presence of Aβ42.

As reported previously, the release of pro-inflammatory cytokines from microglial cells under pathological conditions is a significant event in AD [57]. In the present study, we examined the mRNA expression of different pro-inflammatory cytokines (IL-1β, TNF-α, and IL-6) after treating HMC3 cells with 1 µM Aβ42 for different time intervals. Accordingly, we identified 3 h as a suitable incubation time for the induction of inflammation, as this length of incubation was found to be conducive to the maximal expression of the assessed inflammatory markers. Having established this parameter, we proceeded to repeat the same experiment in the presence and absence of C3G and thus found that C3G can attenuate the Aβ42-induced release of pro-inflammatory cytokines, which, consistent with previous findings [45, 46, 50, 51, 55], provided convincing evidence for the role of C3G as an anti-inflammatory agent.

It has previously been reported that natural flavonoids such as apigenin and chrysin can potentially shift the M1/M2 status of macrophages via activation of PPARγ in mice fed a high-fat diet [58, 59], and the findings of a recent study have revealed that C3G can ameliorate PM10-induced pulmonary injury by modulating M1/M2 macrophage polarization [60]. Also, recent studies have provided the evidences for the anti-inflammatory role of PPARγ in various diseases and in mediating macrophage polarization [61, 62]. In the present study, we found that C3G treatment induced a shift in the phenotype of microglia from M1 to M2 by downregulating the cell surface expression of M1-specific markers (CD80 and CD86), which were found to be increased in the Aβ42-treated group and induced cell surface expression of M2-specific markers (CD206 and CD163).

We also found that C3G induced cell surface expression of PPARγ protein in Aβ42-induced HMC3 cells. Conversely, the presence of the PPARγ antagonist GW9662 was observed to suppress the downregulatory effect of C3G on Aβ42 induction of the classically activated M1 marker CD86. In contrast, upregulation of the alternatively activated M2 marker CD206 was slightly downregulated in the presence of GW9662, although it was not significant. This latter observation, contrasts with the findings of previous studies on flavonoids such as apigenin and chrysin, in which CD206 was found to be significantly downregulated in the presence of GW9662 [58, 59]. These results do, nevertheless, indicate that the C3G-induced microglial shift is mediated via PPARγ.

ROS play a prominent role in the pathogenesis of AD [9–11]. In this regard, the anti-oxidative properties of anthocyanins are well established, and C3G has previously been demonstrated to attenuate Aβ25-35-induced ROS production [13, 50]. Consistently, we found that C3G inhibited Aβ42-induced ROS production in HMC3 cells.

Among the factors contributing to the progression of AD is the limited phagocytosis of Aβ plaques by microglial cells [63]. TREM2 is a well-established phagocytic receptor present on the surface of microglial cells, which has been demonstrated to play a role in anti-inflammatory activities [25–31]. Li et al. recently examined the effects of bilberry anthocyanins in improving neuroinflammation and cognitive impairment in an in vivo model of AD via the CD33/TREM2/TYROBP pathway and found that bilberry could inhibit altered TREM2 expression in the hippocampus [64]. Furthermore, Akhter et al. have described the pivotal role of TREM2 in phagocytosis, inflammation, and apoptosis [30]. In the present study, we found that C3G induced the cell surface expression of TREM2 in Aβ42-treated HMC3 cells and also enhanced the phagocytosis of Aβ42 in these cells, thereby indicating that the upregulated expression of TREM2 is a critical factor contributing to a reduction in Aβ toxicity and enhancement of phagocytosis. Confirmatory evidence in this regard was obtained by pre-treatment with a TREM2-blocking antibody for 1 h prior to the treatment with Aβ and C3G, which abolished the enhanced phagocytosis. Collectively, these findings thus provide compelling evidence to indicate that the enhancement of Aβ42 phagocytosis in HMC3 cells induced by C3G is mediated via TREM2.

Having characterized the effects of C3G in vitro, we went on to examine the activity of this anthocyanin in vivo in an APPswe/PS1ΔE9 mouse model and accordingly obtained consistent results. We found that C3G was able to downregulate mRNA expression of the pro-inflammatory cytokines IL-1β, TNF-α, and IL-6 in the cortex region of the brains of APPswe/PS1ΔE9 mice, which is consistent with the findings of a previous study that established the protective effects of C3G and cyanidin in a colitis mouse model [65]. Similarly, our observations of the enhanced mRNA expression of M2-specific markers CD206 and Arg1 are comparable to those reported previously [60]. Also, similar to the in vitro findings, here also, we found enhanced expression levels of PPARγ and TREM2 in the C3G treated group which were in the close proximity of the previously published studies on flavonoids [58, 64, 66].

To the best of our knowledge, this is the first study to demonstrate the effects of C3G in shifting the M1/M2 polarization of microglia via activation of PPARγ and the TREM2-mediated enhancement of Aβ phagocytosis in Aβ42-treated HMC3 cells. However, further studies, both in vitro and in vivo, are required to establish the precise mechanism underlying the C3G-regulated M1/M2 shift. Furthermore, additional studies are needed to determine the role of phagocytic receptors other than TREM2 present on the microglial cell surface, thereby enabling us elucidate the mechanisms underlying the C3G-medicated phagocytosis of Aβ42.

## Conclusions

In conclusion, in this study, we established the potential utility of the anthocyanin C3G as a therapeutic agent in the treatment of AD, based on our observations of its effects in regulating the M1/M2 status of microglia, in addition to controlling different cellular inflammatory pathways, as previously reported. C3G not only has anti-inflammatory properties but also contributes to eliminating accumulated β-amyloid by enhancing phagocytosis.

## Data Availability

All the data and results obtained during the current study are available from the corresponding author on reasonable request.
