# Correction to: Cyanidin-3-O-Glucoside Regulates the M1/M2 Polarization of Microglia via PPARγ and Aβ42 Phagocytosis Through TREM2 in an Alzheimer’s Disease Model

**DOI:** 10.1007/s12035-023-03877-9

**Published:** 2023-12-22

**Authors:** Sanjay, Jae-Ho Shin, Miey Park, Hae-Jeung Lee

**Affiliations:** 1 Department of Food Science and Biotechnology, College of BioNano Technology, Gachon University, Seongnam-Si 13120, Gyeonggi-Do, Korea; 2 Department of Biomedical Laboratory Science, Eulji University, Gyeonggi-Do 461-713, Seongnam-Si, Republic of Korea; 3 Department of Food and Nutrition, College of BioNano Technology, Gachon University, Seongnam-Si 13120, Gyeonggi-Do, Korea; 4 Institute for Aging and Clinical Nutrition Research, Gachon University, Seongnam-Si 13120, Gyeonggi-Do, Korea

**Correction to: Molecular Neurobiology (2022) 59:5135–5148**

10.1007/s12035-022-02873-9

The original version of this article unfortunately contained mistake.

In Funding section, the grant number is correct however, the corresponding year is incorrect. It should be 2020 instead of 2022 to read “This study was supported by the Cooperative Research Program of the Center for Companion Animal Research (Project No. PJ01476703), funded by the Rural Development Administration, Republic of Korea and Gachon University research fund of 2020 (GCU-202008490009).”

The original article has been corrected.

